# Associations Between Smoking and Cognitive Function Among Community-Dwelling U.S. Chinese Older Adults in Chicago

**DOI:** 10.1093/geroni/igaa057.505

**Published:** 2020-12-16

**Authors:** Shengxi Sun, Nannan Zhang, Mengting Li, XinQi Dong

**Affiliations:** 1 Rutgers, the State University of New Jersey, Chicago, Illinois, United States; 2 East China University of Science and Technology, Shanghai, China; 3 Rutgers University, New Brunswick, New Jersey, United States

## Abstract

Previous studies on smoking and cognition reported mixed findings. The inconsistent results are partially explained by the fact that they were limited to specific populations and using different cognitive function measurements. This association between smoking and cognition has rarely been studied in the rapidly increasing U.S. Chinese older adults. This study aims to determine if smoking status and smoking amount are associated with global cognition and cognitive domains in U.S. Chinese older adults. Data was extracted PINE. Five cognitive function tests (East Boston Memory Test, East Boston Memory Delayed Recall, Digital Backward test, Symbol digit Modality Test, and MMSE) were used to measure cognitive domains including episodic memory, working memory, and processing speed. Five cognitive tests were converted to z scores and averaged to generate global cognition. Self-reported smoking status was used for generating smoking status and smoking amount (pack-years). Linear regression was used. The results showed that former smokers had lower global cognition (b=-0.111, SE=0.053, p<.05) and perceptual speed (b=-0.185, SE=0.066, p<.01) than never smokers; current smokers had lower global cognition (b=-0.240, SE=0.060, p<.001), working memory (b=-0.340, SE=0.083, p<.001) and perceptual speed (b=-0.370, SE=0.075, p<.001) compared with never smokers. Smoking pack-years is negatively associated with global cognition (b=-0.003, SE=0.001,p<.001), episodic memory (b=-0.005, SE=0.001, p<.001), and perceptual speed (b=-0.004, SE=0.001, p<.001). Findings revealed that among all smokers, current smokers had the worst cognition and heavier smoking was associated with worse cognition. Policymakers could take measures in lowering smoking amount among U.S. Chinese older adults to protect their cognition.

